# m^6^A Regulator Expression Segregates Meningiomas Into Biologically Distinct Subtypes

**DOI:** 10.3389/fonc.2021.760892

**Published:** 2021-12-22

**Authors:** Jiawei Chen, Shuchen Sun, Leihao Ren, Lingyang Hua, Daijun Wang, Qing Xie, Hans-Georg Wirsching, Jiaojiao Deng, Michael Weller, Ye Gong

**Affiliations:** ^1^ Department of Neurosurgery, Huashan Hospital, Fudan University, Shanghai, China; ^2^ Institute of Neurosurgery, Fudan University, Shanghai, China; ^3^ Shanghai Key Laboratory of Brain Function Restoration and Neural Regeneration, Fudan University, Shanghai, China; ^4^ Department of Neurology, University Hospital and University of Zurich, Zurich, Switzerland; ^5^ Department of Critical Care Medicine, Huashan Hospital, Fudan University, Shanghai, China

**Keywords:** meningioma, immune infiltration, m^6^A, WGCNA, molecular subtype

## Abstract

**Background:**

Meningiomas are the most common primary intracranial tumors in adults. According to the 2021 World Health Organization (WHO) classification of central nervous system tumors, approximately 80% of meningiomas are WHO grade 1, that is, histopathologically benign, whereas about 20% are WHO grade 2 or grade 3, showing signs of atypia or malignancy. The dysregulation of N6-methylation (m^6^A) regulators is associated with disorders of diverse critical biological processes in human cancer. This study aimed to explore whether m^6^A regulator expression was associated with meningioma molecular subtypes and immune infiltration.

**Methods:**

We evaluated the m^6^A modification patterns of 160 meningioma samples based on 19 m^6^A regulators and correlated them with immune infiltration characteristics. Novel molecular subtypes were defined based on prognostic hub gene expression.

**Results:**

Two meningioma clusters were identified based on the expression of 19 m^6^A regulators. In cluster 1, 607 differentially expressed genes (DEGs) were upregulated and 519 were downregulated. A total of 1,126 DEGs comprised three gene expression modules characterized by turquoise, blue, and gray. Functional annotation suggested that the turquoise module was involved in Wnt-related and other important cancer-related pathways. We identified 32 hub genes in this module by constructing a protein–protein interaction network. The meningioma samples were divided into two molecular subtypes. *EPN1*, *EXOSC4*, *H2AX*, and *MZT2B* not only showed significant differences between meningioma molecular subtypes but also had the potential to be the marker genes of specific meningioma subtypes.

**Conclusion:**

m^6^A regulator gene expression may be a novel prognostic marker in meningioma.

## Introduction

Meningiomas arise from arachnoid cap cells attached to the inner layer of the dura, which covers the spinal cord and brain. They represent about 37.6% of primary central nervous system tumors, making them the most common histological types of intracranial tumor, with an incidence of 8.83 per 100,000 (1). They primarily occur in elderly individuals, with increased incidence in individuals older than 65 years (1). The incidence of meningiomas has also increased among adolescents and young adults; these tumors now represent about 16% of all intracranial tumors in people aged 15–39 years (2). Meningiomas preferentially affect women, with a female-to-male ratio between 2:1 and 3.5:1 (3–5). According to the 2021 World Health Organization (WHO) classification of central nervous system tumors, approximately 80% of cases are WHO grade 1 meningiomas with benign histology, whereas about 20% of cases are WHO grade 2 and 3 meningiomas showing signs of increased malignancy at histology (6).

RNA methylation, including 5-methylcytosine (m^5^C), N6-methyladenosine (m^6^A), and N1-methyladenosine (m^1^A), has become a common phenomenon and a critical regulating factor for transcript expression in different types of cancer (7, 8). N6-methylation (m^6^A), methylated at the N6 position of adenosine, has been regarded as the most pervasive, abundant, and conserved internal transcriptional modification within eukaryotic messenger RNAs (mRNAs), microRNAs (miRNAs), and long non-coding RNAs (lncRNAs) (9, 10). The deposition of m^6^A is encoded by a methyltransferase complex involving three homologous factors: methyltransferases (termed as “writers”), demethylases (termed as “erasers”), and recognition from m^6^A-binding proteins (termed as “readers”) (11). The m^6^A dysregulation, caused by dysregulated expression and genetic changes in m^6^A regulators, is related to the disorders of multiple critical biological processes in human cancer (12, 13). Qi et al. reported that the self-renewal and tumorigenesis of glioma stem cells (GSCs) were regulated by m^6^A RNA methylation, and an m^6^A mRNA demethylase FTO inhibitor could suppress the progression of GSC-initiated tumor (14). Yang et al. demonstrated that FTO played an important role in promoting melanoma tumorigenesis and anti-PD-1 resistance, and the combination of FTO inhibitors with anti-PD-1 blockers could reduce the resistance to immunotherapy in melanoma (15). Miao et al. revealed that m^6^A methyltransferase METTL3 promoted osteosarcoma cell progression by regulating the m^6^A level of LEF1 and activating the Wnt/β-catenin signaling pathway (16). Besides, Vengoechea J. et al. observed that IGF2BP1, one of the m^6^A regulators, could increase the malignant potential of meningiomas by enhancing cell adhesion (17). Hwang M. et al. identified significantly higher expression of HNRNPA2B1 in benign meningioma compared to normal brain tissue (18).

Numerous studies revealed that the tumor microenvironment (TME) was fundamental for tumor survival, growth, and progression. The immune part of TME contained tumor-associated macrophages, tumor-associated neutrophils, dendritic cells, myeloid-derived suppressor cells (MDSCs), and Tie2-expressing monocytes comprising tumor-associated myeloid cells (19). Patients with meningiomas exhibit signs of peripheral immunosuppression, including increased PD-L1 on myeloid cells and elevated MDSC abundance proportional to tumor grade (20). The accumulation of mast cells in meningiomas could contribute to the aggressiveness of tumors (21). Chen et al. revealed that the proportions of tumor-infiltrating immune cells were associated with the prognosis for patients with meningioma (22). Overall, tumor cells elicited multiple biological behavioral changes through direct and indirect interactions with immune cells, such as inducing proliferation and angiogenesis, inhibiting apoptosis, avoiding hypoxia, and inducing immune tolerance (23–26). Therefore, a deeper understanding of the immune infiltration of meningiomas could help parse the TME landscape and finding promising biomarkers for immunotherapy. However, whether immune infiltration in meningiomas is regulated through the modification of m^6^A patterns is still unknown. To address this question, we established a meningioma classification based on m^6^A regulator gene expression and evaluated the associations of m^6^A-deduced subtypes with immune infiltration in meningioma.

## Materials and Methods

### Meningioma Dataset Resource and Processing

The Gene Expression Omnibus (GEO) database, restoring high-throughput gene expression data and hybridization arrays, chips, and microarrays, allows an easy access to gene expression data of human cancer. Public gene expression data and related clinical annotation data were obtained from the GEO database. GSE136661 and GSE43290 were gathered in the present study for further analysis (**Table 1**) (27, 28). The expression dataset GSE136661 with 160 meningioma samples from the Illumina HiSeq 4000 platform and GSE43290 with 47 meningioma samples and 4 normal meningeal samples from the Affymetrix Human Genome U133A Array platform were downloaded from the GEO database. Multiple probes corresponding to a gene were retained and shown as the median of the gene expression level, while probes corresponding to multiple genes were eliminated. The clinical information was also extracted from GEO raw data.

**Table 1:** Meningioma gene expression data from GEO database.

Dataset ID	Platform	Samples
GSE136661	GPL20301	160
GSE43290	GPL96	51

### m^6^A Regulator Data Retrieval From GEO Datasets

According to Zhang et al., 21 m^6^A regulators were extracted from 5 integrated GEO datasets, including 8 writers (METTL3, METTL14, RBM15, RBM15B, WTAP, KIAA1429, CBLL1, and ZC3H13), 2 erasers (ALKBH5 and FTO), and 11 readers (YTHDC1, YTHDC2, YTHDF1, YTHDF2, YTHDF3, IGF2BP1, HNRNPA2B1, HNRNPC, FMR1, LRPPRC, and ELAVL1) (29). After intersecting with the GSE136661 dataset, 19 m^6^A regulators were included for further analysis, except for RBM15B and KIAA1429.

### Differentially Expressed Genes Screening

Data analysis was performed using the limma package and *t* test. Fold change > 2.0 or <0.5 and *P <*0.05 were defined as cutoffs to screen for differentially expressed genes (DEGs) between different m^6^A clusters.

### Immune Cell Infiltration Analysis

CIBERSORT (https://cibersort.stanford.edu/index.php) was employed to characterize cell composition based on the gene expression profiles of complex tissues (30). A white blood cell gene matrix (LM22) consisting of 547 genes was used to identify 22 immune cell types, including myeloid subsets, natural killer cells, plasma cells, naive and memory B cells, and T cells. CIBERSORT was combined with the LM22 eigenmatrix to estimate the proportions of 22 immune cell phenotypes in different m^6^A cluster 1 and m^6^A cluster 2.

### Weighted Gene Co-Expression Network Construction and Hub Gene Screening

The expression profile of the aforementioned DEGs was obtained to establish a gene co-expression network by the weighted gene co-expression network analysis (WGCNA) package in R (Version 4.1.0). The threshold power of *β* was used for constructing co-expression modules based on size independence and average connectivity of modules. We built a scale-free topology by underlining the strong correlations and attenuating the weak correlations with the soft threshold power of *β* = 3 (scale-free *R* ^2^ = 0.85). Then, the topological overlap matrix was calculated based on adjacency matrices. We applied the dynamic tree cut algorithm to classify genes according to their expression patterns and merged gene modules (at least 30 genes were included). The module eigengene (ME) was calculated as a summary profile for all genes in a module. These modules were merged into three major modules (blue, gray, and turquoise) by clustering analysis. We calculated the Pearson correlation coefficient of these three modules and m^6^A cluster characteristics and selected the most correlated module for further analysis. Gene significance (GS) was employed as the correlation coefficient between transcriptome expression and module traits. Module significance was defined as the correlation coefficient between the module and the traits. Module membership (MM) was defined by the correlation coefficient of the ME and transcriptome data. Genes with a GS >0.60 and MM >0.80 were selected as each module’s candidate hub genes. STRING is a database of known and predicted protein–protein interactions. The interactions include direct (physical) and indirect (functional) associations; they stem from computational prediction, from knowledge transfer between organisms, and from interactions aggregated from other (primary) databases. (30476243) A protein–protein interaction (PPI) network contained all candidate hub genes obtained from the STRING database. Nodes with 10 or more edges in the PPI network were selected to intersect with the module’s candidate hub genes, and the key hub genes were finally identified.

### Functional Enrichment Analysis

The functional enrichment analysis of DEGs was performed to identify Gene Ontology categories by their biological processes, molecular functions (MF), and cellular components and Kyoto Encyclopedia of Genes and Genomes (KEGG) enrichment analyses using the DAVID tool (https://david.ncifcrf.gov/tools.jsp) (31).

### Unsupervised Clustering for Meningioma m^6^A Clusters and Molecular Subtypes

Meningioma m^6^A clusters and molecular subtypes were determined according to the expression profile of m^6^A regulators and hub genes, respectively. The best K value (number of categories) was determined by finding the optimal sum of the squared error (SSE). The meningioma samples were divided into different subtypes by unsupervised clustering K-means and *t*-Distributed Stochastic Neighbor Embedding (t-SNE) descending dimension method. Significant DEGs, which might potentially become the marker genes of meningioma, were identified between different molecular subtypes using the Kruskal−Wallis test (*P* < 0.05).

### Statistics Analysis

The expression levels of DEGs higher and lower than the median value were considered high and low expression levels, respectively. The Kruskal–Wallis test was used to conduct different comparisons of three or more groups. All statistical *P* values were two-sided, with *P <*0.05 indicating statistically significance. All data processing was done in R software (Version 4.1.0).

## Results

**Figure 1:**
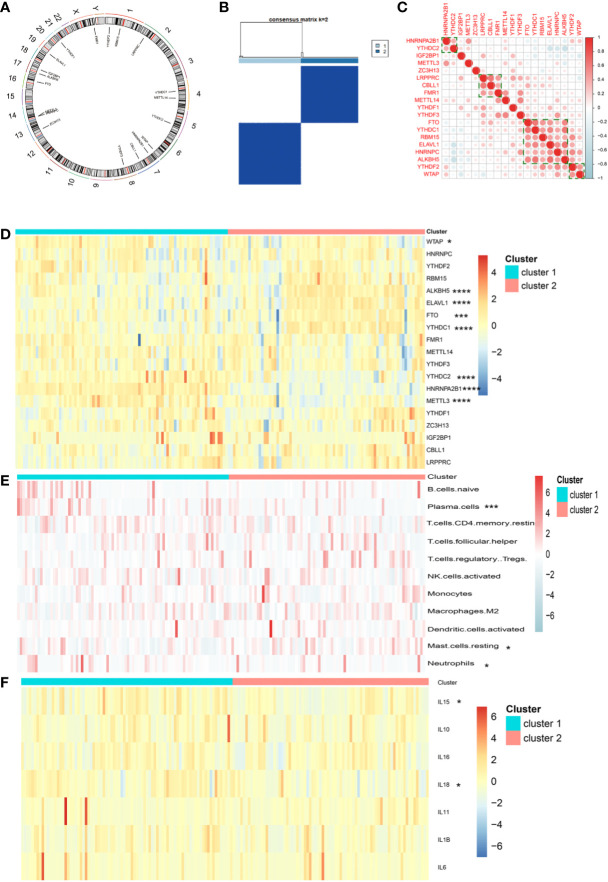
**(A)** Location of 19 m^6^A regulators on 23 chromosomes using GSE136661 cohort. **(B)** Clustering of meningioma samples. **(C)** Correlations between among 19 m^6^A regulators in the GSE136661 cohort using Spearman analysis. Negative correlation was marked with blue and positive correlation with red. **(D)** Heatmap of the expression of 19 m^6^A regulators in two distinct m^6^A clusters. **(E)** Heatmap of immune cell infiltration in two distinct m^6^A clusters. **(F)** Heatmap of the expression of inflammatory reaction-related genes in two distinct m^6^A clusters (^*^ *P* < 0.05; ^***^ *P* < 0.005; ^****^ *P* < 0.001).

A total of 19 m^6^A regulators were identified in the present study, including 6 writers, 2 erasers, and 11 readers. The genes of these regulators were distributed widely on multiple human chromosomes (**Figure 1A**). Several m^6^A regulators were co-expressed, including (a) HNRNPA2B1 and YTHDC2, (b) LRPPRC, CBLL1, and FMR1, (c) FTO, YTHDC1, RMB15, ELAVL1, HNRNPC, and ALKBH5, and (d) YTHDF2 and WTAP (**Figure 1C**). K-means unsupervised clustering based on the expression of the 19 m^6^A regulators segregated meningioma samples into 2 clusters (**Figure 1B** and **Supplementary Figures 1A, B**). PCA showed that these two different m^6^A clusters could be well separated (**Supplementary Figure 1C**). Eight m^6^A regulators were differentially expressed between both clusters, including WTAP, ALKBH5, ELAVL1, FTO, YTHDC1, YTHDC2, HNRNPA2B1, and METTL3 (*P* < 0.05, **Figure 1D**). Employing CIBERSORT to estimate immune infiltration in both clusters suggested different numbers of plasma B cells, resting mast cells, and neutrophils in the two m^6^A clusters (*P* < 0.05, **Figure 1E**). As for some major inflammatory reaction−related genes, we found that the expression of IL-15 and IL-18 was also significantly different between the two distinct m^6^A clusters (*P* < 0.05, **Figure 1F**).

**Figure 2:**
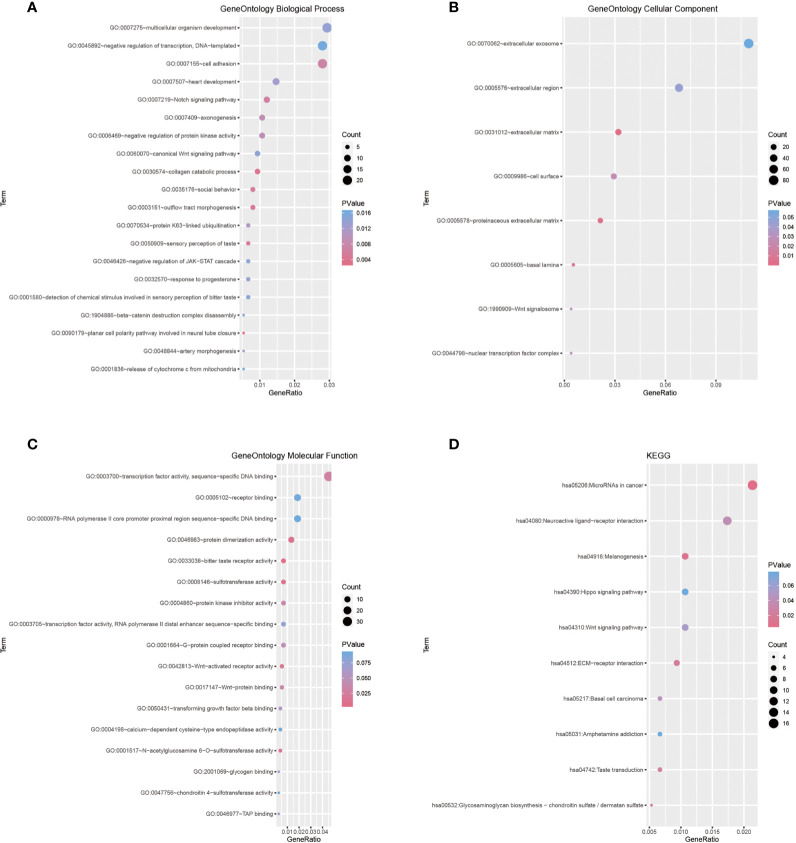
**(A–C)** Gene Ontology terms in the biological process, cellular component, and molecular function categories. **(D)** Enrichment plot conducted *via* KEGG analysis.

Next, we performed weighted gene co-expression network analyses to better characterize the biological traits of both clusters. A total of 1,126 DEGs were used for the construction of weighted gene co-expression networks. A soft threshold power of *β* = 3 was used to construct co-expression modules (**Supplementary Figures 2A, B**). Three modules were identified and designated as the blue, gray, and turquoise modules, comprising 137, 179, and 810 genes, respectively (**Supplementary Figure 2C**). WGCNA was then applied to explore the module-trait relationships of different modules and m^6^A clusters, and the turquoise module was identified as a hub gene set for explaining the difference between both clusters (**Supplementary Figure 2D**). Gene set enrichment analysis of the 810 genes in the turquoise module identified Notch and Wnt signaling as the key differentially expressed oncogenic pathways in either cluster (**Figures 2A**–**D**).

**Figure 3:**
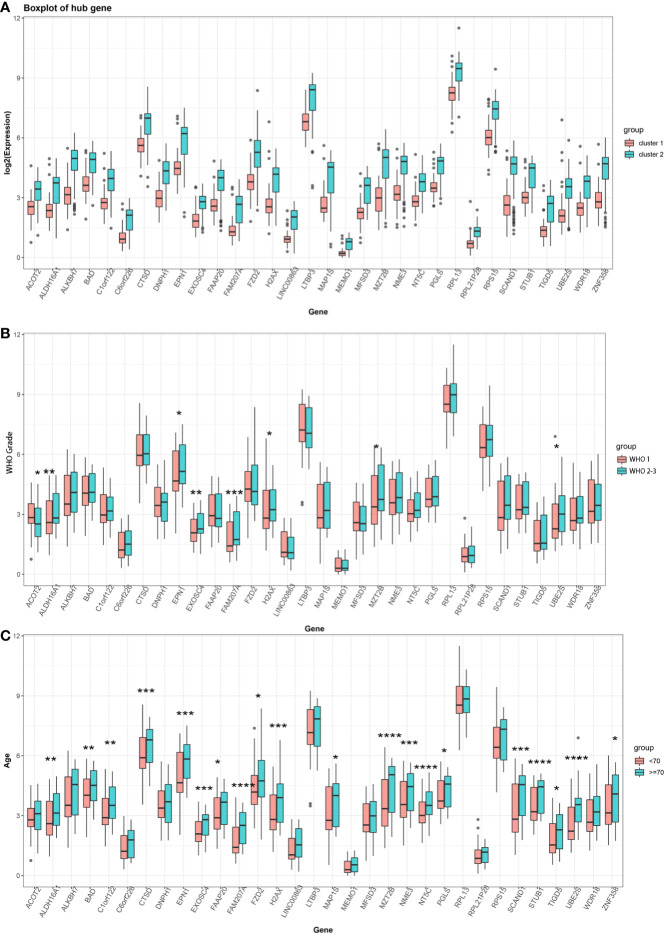
**(A)** Expression of 32 key hub genes in two distinct m^6^A clusters. **(B)** Expression of 32 key hub genes in different meningioma WHO grades (Grade 1 *vs* Grade 2–3). **(C)** Expression of 32 key hub genes at different ages of patients with meningioma (<70 years old *vs* ≥70 years old). (^*^ *P* < 0.05; ^**^ *P* < 0.01; ^***^ *P* < 0.005; ^****^ *P* < 0.001).

We chose a combined approach by (i) employing the STRING tool for functional interaction analyses to identify key network nodes (**Supplementary Figure 3A**) and (ii) calculating the Pearson correlation coefficient of the turquoise module and m^6^A cluster characteristics to define hub genes and further characterize each m^6^A cluster (**Supplementary Figure 3B**). After intersecting these two results, 32 key hub genes were identified (**Supplementary Figure 3C**). These key hub genes comprised *ACOT2*, *ALDH16A1*, *ALKBH7*, *BAD*, *C1orf122*, *C6orf226*, *CTSD*, *DNPH1*, *EPN1*, *EXOSC4*, *FAAP20*, *FAM207A*, *FZD2*, *H2AX*, *LINC00863*, *LTBP3*, *MAP1S*, *MEMO1*, *MFSD3*, *MZT2B*, *NME3*, *NT5C*, *PGLS*, *RPL13*, *RPL21P28*, *RPS15*, *SCAND1*, *STUB1*, *TIGD5*, *UBE2S*, *WDR18*, and *ZNF358.* All of these 32 hub genes had lower expression in cluster 1 and higher expression in cluster 2 (*P* < 0.05, **Figure 3A**). Moreover, 8 hub genes were associated with WHO grade (**Figure 3B**) and 20 were associated with age (**Figure 3C**), but no gene was related to the sex of patients with meningioma.

**Figure 4:**
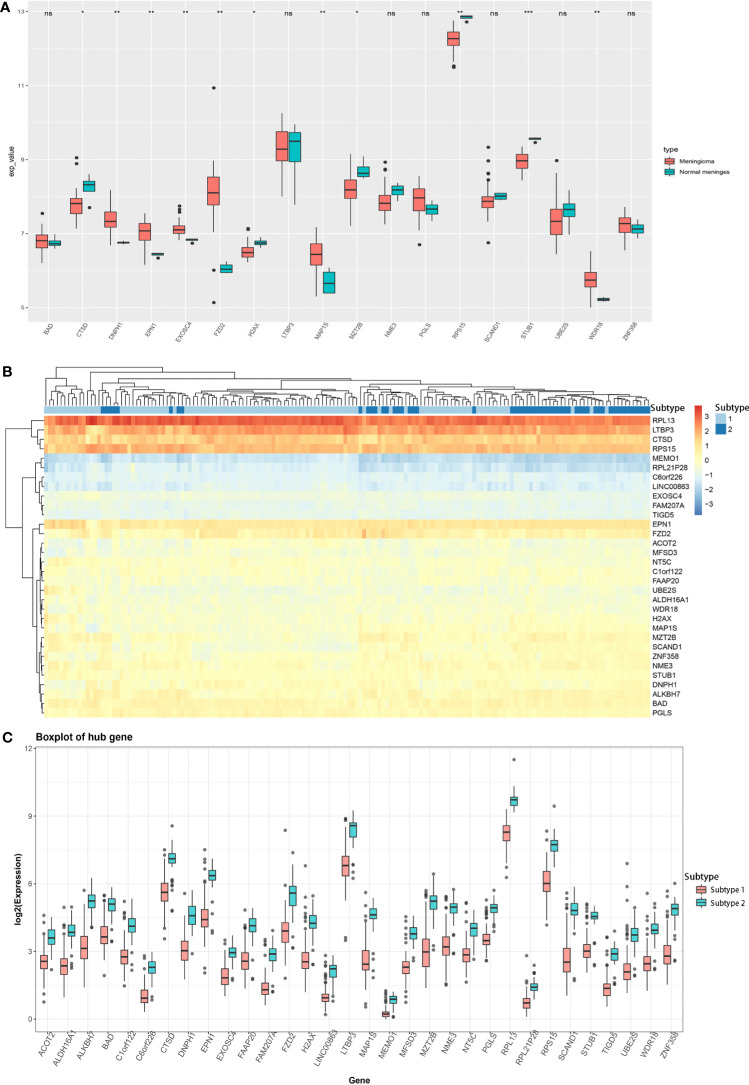
**(A)** Expression of 18 key hub genes between normal meningeal tissues and meningioma tissues. **(B)** Heatmap of the expression of 32 key hub genes between two different meningioma molecular subtypes. **(C)** Expression of 32 key hub genes in two different meningioma molecular subtypes. (^*^ *P* < 0.05; ^**^ *P* < 0.01; ^***^ *P* < 0.005; ns, Non-significant.

Besides, we also analyzed the association of key hub genes with normal meningeal tissues and meningiomas. Further, 18 out of 32 key hub genes were included in the GSE43290 dataset, and 11 key hub genes (*CTSD*, *DNPH1*, *EPN1*, *EXOSC4*, *FZD2*, *H2AX*, *MAP1S*, *MZT2B*, *RPS15*, *STUB1*, and *WDR18*) showed significantly different expression between normal meningeal tissues and meningiomas (*P* < 0.05, **Figure 4A**).

Based on the expression profile of these 32 key genes, we divided the meningioma samples into two clusters by K-means unsupervised clustering (**Supplementary Figures 4A, B**). Based on the heatmap drawn by R language, the combination of these 32 key hub genes could help distinguish the meningioma dataset into two subtypes, indicating that these 32 key hub genes were critical for meningioma molecular subtypes (**Figure 4B**). Furthermore, we analyzed the expression of these 32 key hub genes and discovered that all these 32 key hub genes had significantly different expression levels between two different molecular subtypes. Also, all of them had lower expression in subtype 1 and higher expression in subtype 2, which uncovered that these key hub genes might function as the marker genes of different meningioma molecular subtypes (**Figure 4C**).

**Figure 5:**
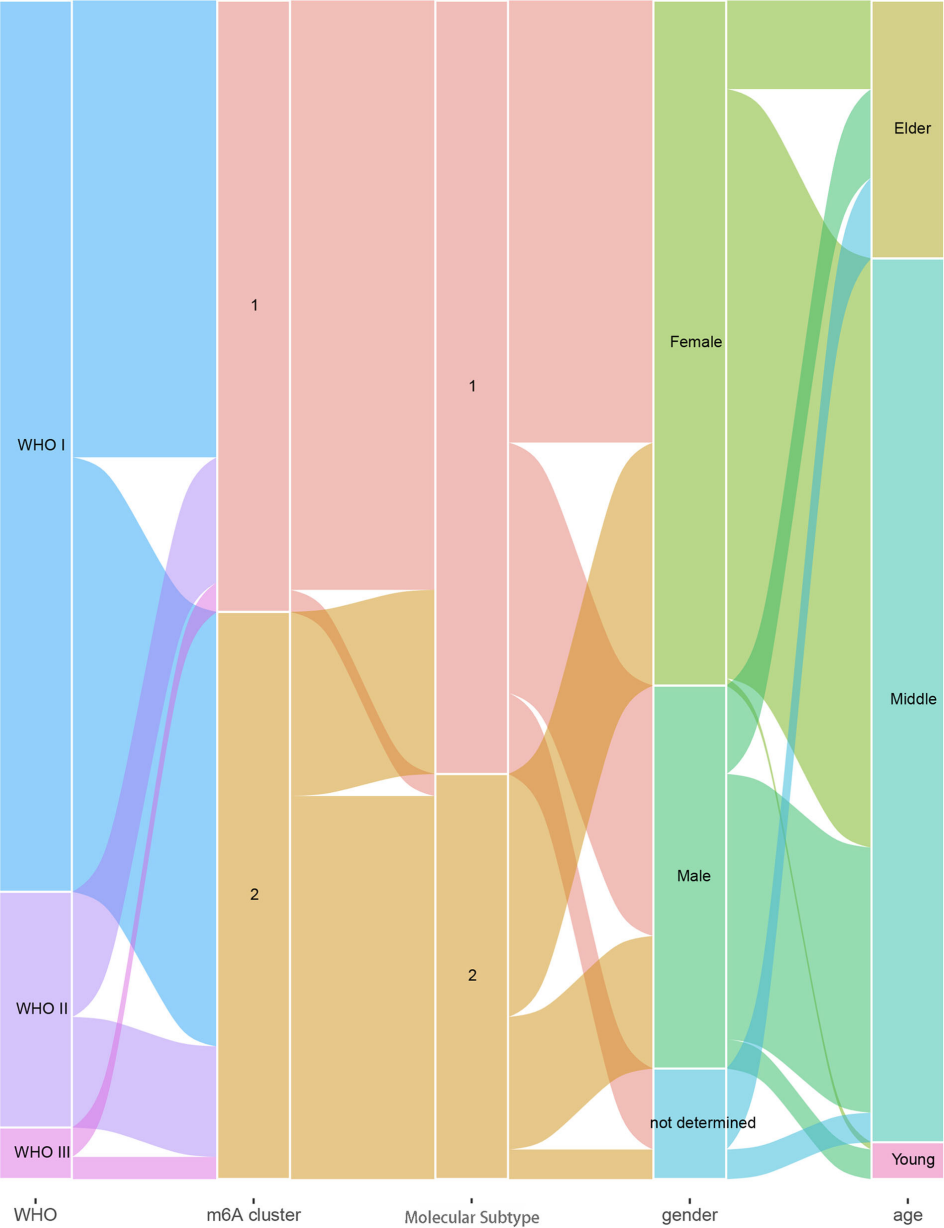
Relation diagram of meningioma WHO grades, m^6^A clusters, molecular subtypes, sex, and age.

For a better understanding of the association of m^6^A clusters and molecular subtypes with the sex, age, and WHO grade of patients with meningioma, we conducted a correlation analysis and found that every WHO grade was composed of two m^6^A clusters and two molecular subtypes, and the age of patients was most likely between 30 and 70 years (**Figure 5**).

## Discussion

Increasing evidence revealed that m^6^A modification played an important role in regulating tumor immunity and shaping TME through interaction with various m^6^A regulators. However, most research focused on a single m^6^A regulator, and the integrated roles of multiple m^6^A regulators were not comprehensively understood, especially for meningioma.

The present study investigated the association of meningioma with multiple m^6^A regulators, established meningioma classification based on m^6^A regulator gene expression, and evaluated its merit with immune infiltration in meningioma. Based on 19 m^6^A regulators, we constructed and segregated meningiomas into two clusters. We found that the genes of 19 m^6^A regulators distributed widely on multiple human chromosomes and different m^6^A regulators [e.g., (a) HNRNPA2B1 and YTHDC2; (b) LRPPRC, CBLL1, and FMR1; (c) FTO, YTHDC1, RMB15, ELAVL1, HNRNPC, and ALKBH5; and (d) YTHDF2 and WTAP] might have common effects. As for the difference between two distinct m^6^A clusters, we revealed that the gene expression of WTAP, ALKBH5, ELAVL1, FTO, YTHDC1, YTHDC2, HNRNPA2B1, and METTL3; the infiltration of some immune cells (plasma cells, resting mast cells, and neutrophils); and the expression of IL15 and IL18 were significantly different. A total of 1,126 DEGs were included in their different modules between the two different m^6^A clusters, and the turquoise module containing 810 DEGs was the key module. Besides, these 810 DEGs played regulatory roles in organ and tissue generation and development and multiple important signaling pathways involved in tumor development. *ACOT2*, *ALDH16A1*, *ALKBH7*, *BAD*, *C1orf122*, *C6orf226*, *CTSD*, *DNPH1*, *EPN1*, *EXOSC4*, *FAAP20*, *FAM207A*, *FZD2*, *H2AX*, *LINC00863*, *LTBP3*, *MAP1S*, *MEMO1*, *MFSD3*, *MZT2B*, *NME3*, *NT5C*, *PGLS*, *RPL13*, *RPL21P28*, *RPS15*, *SCAND1*, *STUB1*, *TIGD5*, *UBE2S*, *WDR18*, and *ZNF358* were identified as key hub genes, and all these genes could be the marker genes to distinguish different m^6^A clusters for their different expression. Among these DEGs, the expression of *FAM207A*, *EXOSC4*, *ALDH16A1*, *MZT2B*, *UBE2S*, *ACOT2*, *EPN1*, and *H2AX* was significantly related to WHO grades, while the expression of *UBE2S*, *FAM207A*, *NT5C*, *STUB1*, *MZT2B*, *SCAND1*, *EPN1*, *CTSD*, *EXOSC4*, *H2AX*, *NME3*, *ALDH16A1*, *C1orf122*, *BAD*, *TIGD5*, *MAP1S*, *FAAP20*, *FZD2*, *PGLS*, and *ZNF358* was significantly related to the age of patients with meningioma. Additionally, *CTSD*, *DNPH1*, *EPN1*, *EXOSC4*, *FZD2*, *H2AX*, *MAP1S*, *MZT2B*, *RPS15*, *STUB1*, and *WDR18* showed significantly different expression levels between normal meningeal tissues and meningiomas. Furthermore, the combination of these 32 DEGs could be marker genes to help in segregating meningiomas into 2 subtypes based on their gene expression. The expression of *EPN1*, *EXOSC4*, *H2AX*, and *MZT2B* was related to both WHO grades and age of patients and was significantly different between normal meningeal tissues and meningiomas.

Several studies revealed the importance of the m^6^A modification pattern, regulated by m^6^A regulators, for the development and progression of a tumor. Liu et al. revealed reduced m^6^A mRNA methylation as an oncogenic mechanism in endometrial cancer and identified m^6^A methylation as a regulator of Akt signaling (32). However, Li et al. found enhanced m^6^A mRNA methylation as an oncogenic mechanism in hepatoblastoma because METTL3 was significantly upregulated and promoted hepatoblastoma development (33). Du et al. suggested two distinct m^6^A modification patterns (an immune-activated differentiation pattern and an immune-desert dedifferentiation pattern) in lower-grade glioma, which were associated with different clinical outcomes, burden of neoepitope, immune infiltration, and stemness (34). The emerging functions of m^6^A regulators in GSCs and immune infiltration have been confirmed, including roles in radio-chemotherapy resistance, tumorigenesis, promotion of the self-renewal of cancer stem cells, programmed proliferation of cancer cells, induction of apoptosis, and reduction of migration (8, 35, 36). Xu et al.’s work demonstrated the carcinogenic activity of FTO in promoting the invasion and migration of breast cancer cells *via* the FTO/miR-181b-3p/ARL5B signaling pathway, which highlighted the important role of FTO in tumor pathogenesis (37). Chang et al. uncovered an essential role of YTHDF3 in regulating the interaction between breast cancer cells and brain microenvironment by upregulating key brain metastatic proteins, thereby facilitating brain metastasis (38). Wang et al.’s research suggested that the upregulation of METTL14 could lead to the decrease of PERP mRNA levels *via* m^6^A modification, promoting the growth, invasion, and metastasis of pancreatic cancer cells (39). Our results were consistent with the former findings. The m^6^A regulators played an important role in meningiomas and segregated them into two distinct m^6^A clusters, which were correlated with different m^6^A regulator gene expression, interleukin gene expression, and immune cell infiltration. Besides, Mathoux et al. elaborated that m^6^A was enriched in the brain and emerged as a key regulator of neuronal activity and function in processes including neurodevelopment, learning and memory, synaptic plasticity, and stress response (40). Li et al. revealed that YTHDF2, an m^6^A regulator, functioned as a contributor to lung adenocarcinoma development through the upregulation of the AXIN1/Wnt/β-catenin signaling pathway (41). Another study revealed that m^6^A mRNA methylation contributed significantly to regulate the Wnt/β-catenin pathway (33). Similar to the research listed earlier, our present study also found that m^6^A modification patterns were related to several important signaling pathways in meningioma, such as Wnt signaling pathway, and development of organs and tissues in the nervous system.

With the rapid development of multi-omics and big data analysis, more research focuses on meningioma molecular subtypes. DNA methylation-based classification and grading system for meningioma had a higher power for tumor recurrence and progression prediction compared with the WHO classification (42). Another study demonstrated a highly distinct epigenetic signature of clear cell meningiomas, which was associated with frequent mutations within the SMARCE1 gene and/or loss of SMARCE1 protein expression (43). Zador et al. found that WHO grade II meningiomas could be further segregated into two distinct subgroups (a benign “grade I-like” and a malignant “grade III-like”) with different tumor recurrence rates (0 and 75%, respectively) (44). Williams et al. found that the patterns of genomic alterations in high-grade/progressive meningiomas were commonly grouped into three different categories. The NF2-associated canonical group frequently harbored CDKN2A/B alterations, which was potentially amenable to targeted therapies. An NF2-agnostic group harbored frequent TERTp and TP53 mutations. An NF2-exclusive group was partly characterized by BAP1/PBRM1 alterations (rhabdoid/papillary histology) or skull-base disease (45). Also, it was of great importance to identify some marker genes to help distinguish the molecular subtypes of meningioma. EPN1 was identified as one of the hub genes in pediatric medulloblastoma by multiple-microarray analysis (46). EXOSC4 functioned as a potential oncogene in the development and progression of colorectal cancer and was identified as a potential diagnostic molecular biomarker (47). To the best of our knowledge, *EPN1*, *EXOSC4*, *H2AX*, and *MZT2B* have not been identified as the marker genes of specific meningioma subtype.

This study had several limitations. First, the dataset we analyzed lacked survival information, and our results might have been affected by the small sample size. Therefore, improving the sample size, sequencing data, and clinical information of patients with meningioma is of great necessity in further studies. In addition, our results and conclusions are based on the bioinformatics analysis of datasets, which require further verification by basic biological experiments and clinical research.

## Conclusions

In conclusion, this study suggested that the m^6^A regulators played an important role in meningiomas and segregated them into two distinct m^6^A clusters, which were correlated with different m^6^A regulator gene expression, interleukin gene expression, and immune cell infiltration. Also, m^6^A modification patterns were related to several important signaling pathways in meningioma and the development of organs and tissues in the nervous system. Among 32 key hub genes screened, *EPN1*, *EXOSC4*, *H2AX*, and *MZT2B* not only showed significant differences between meningioma molecular subtypes but also had the potential to be the marker genes of specific meningioma subtype.

## Data Availability Statement

Publicly available datasets were analyzed in this study. This data can be found here: https://www.ncbi.nlm.nih.gov/geo/query/acc.cgi?acc=GSE136661 https://www.ncbi.nlm.nih.gov/geo/query/acc.cgi?acc=GSE43290.

## Author Contributions

JC and YG designed the study. SS, LR, DW, and QX obtained and processed the data from GEO database. JC, SS, LH, and JD performed the data analysis. JC, HW, and MW prepared this manuscript. JD managed the research work in the subsequent revision. All authors contributed to the article and approved the submitted version.

## Funding

This study was supported by grants from the National Key R&D Program of China (2018YFC1312600 and 2018YFC1312604 to YG), National Natural Science Foundation of China (82072788 and 81772674 to YG), Shanghai Sailing Program (20YF1403900 to LH).

## Conflict of Interest

The authors declare that the research was conducted in the absence of any commercial or financial relationships that could be construed as a potential conflict of interest.

## Publisher’s Note

All claims expressed in this article are solely those of the authors and do not necessarily represent those of their affiliated organizations, or those of the publisher, the editors and the reviewers. Any product that may be evaluated in this article, or claim that may be made by its manufacturer, is not guaranteed or endorsed by the publisher.

## Supplementary Material

Supplementary Figure 1
**(A, B)** Best inflection point identified by the sum of squares for the error method. **(C)** Principal component analysis of two m^6^A clusters of the GSE136661 cohort.

Supplementary Figure 2
**(A, B)** Analysis of network topology for various soft-thresholding powers. **(C)** Gene dendrogram and module colors. **(D)** Module–trait relationships of different modules and m^6^A clusters.

Supplementary Figure 3
**(A)** PPI network of the turquoise module obtained from the STRING database. **(B)** Correlation analysis of the turquoise module and m^6^A cluster characteristics. **(C)** Venn diagram of central nodes in PPI network and candidate genes in the turquoise module.

Supplementary Figure 4
**(A)** Best inflection point identified by the SSE method. **(B)** Dimensionality reduction and clustering of meningioma samples conducted by the t-SNE algorithm.

## References

[1] Ostrom QT Cioffi G Gittleman H Patil N Waite K Kruchko C. CBTRUS Statistical Report: Primary Brain and Other Central Nervous System Tumors Diagnosed in the United States in 2012-2016. Neuro Oncol (2019) 21:v1–v100. doi: 10.1093/neuonc/noz150 31675094 PMC6823730

[2] Ostrom QT Gittleman H de Blank PM Finlay JL Gurney JG McKean-Cowdin R. American Brain Tumor Ass Ociation Adolescent and Young Adult Primary Brain and Central Nervous System Tumors Diagnosed in the United States in 2008-2012. Neuro Oncol (2016) 18 Suppl 1:i1–i50. doi: 10.1093/neuonc/nov297 26705298 PMC4690545

[3] Grunberg SM Weiss MH Spitz IM Ahmadi J Sadun A Russell CA. Treatment of Unresectable Meningiomas With the Antiprogesterone Agent Mifepristone. J Neurosurg (1991) 74:861–6. doi: 10.3171/jns.1991.74.6.0861 2033444

[4] Rohringer M Sutherland GR Louw DF Sima AA. Incidence and Clinicopathological Features of Meningioma. J Neurosurg (1989) 71:665–72. doi: 10.3171/jns.1989.71.5.0665 2809720

[5] Klaeboe L Lonn S Scheie D Auvinen A Christensen HC Feychting M. Incidence of Intracranial Meningiomas in Denmark, Finland, Norway and Sweden, 1968-1997. Int J Cancer (2005) 117:996–1001. doi: 10.1002/ijc.21255 15986431

[6] Louis DN Perry A Wesseling P Brat DJ Cree IA Figarella-Branger D. The 2021 WHO Classification of Tumors of the Central Nervous System: A Summary. Neuro Oncol (2021) 23(8):1231–51. doi: 10.1093/neuonc/noab106 PMC8328013 34185076

[7] Roundtree IA Evans ME Pan T He C. Dynamic RNA Modifications in Gene Expression Regulation. Cell (2017) 169:1187–200. doi: 10.1016/j.cell.2017.05.045 PMC5657247 28622506

[8] Lan Q Liu PY Haase J Bell JL Huttelmaier S Liu T. The Critical Role of RNA M(6)A Methylation in Cancer. Cancer Res (2019) 79:1285–92. doi: 10.1158/0008-5472.CAN-18-2965 30894375

[9] Sample A He YY. Mechanisms and Prevention of UV-Induced Melanoma. Photodermatol Photoimmunol Photomed (2018) 34:13–24. doi: 10.1111/phpp.12329 28703311 PMC5760354

[10] Chen XY Zhang J Zhu JS. The Role of M(6)A RNA Methylation in Human Cancer. Mol Cancer (2019) 18:103. doi: 10.1186/s12943-019-1033-z 31142332 PMC6540575

[11] Lin Y Wang S Liu S Lv S Wang H Li F. Identification and Verification of Molecular Subtypes With Enhanced Immune Infiltration Based on M6a Regulators in Cutaneous Melanoma. BioMed Res Int (2021) 2021:2769689. doi: 10.1155/2021/2769689 33490266 PMC7801086

[12] Tong J Cao G Zhang T Sefik E Amezcua Vesely MC Broughton JP. M(6)A mRNA Methylation Sustains Treg Suppressive Functions. Cell Res (2018) 28:253–6. doi: 10.1038/cr.2018.7 PMC5799823 29303144

[13] Pinello N Sun S Wong JJ. Aberrant Expression of Enzymes Regulating M(6)A mRNA Methylation: Implication in Cancer. Cancer Biol Med (2018) 15:323–34. doi: 10.20892/j.issn.2095-3941.2018.0365 PMC6372906 30766746

[14] Cui Q Shi H Ye P Li L Qu Q Sun G. M(6)A RNA Methylation Regulates the Self-Renewal and Tumorigenesis of Glioblastoma Stem Cells. Cell Rep (2017) 18:2622–34. doi: 10.1016/j.celrep.2017.02.059 PMC5479356 28297667

[15] Yang S Wei J Cui YH Park G Shah D Deng Y. M(6)A mRNA Demethylase FTO Regulates Melanoma Tumorigenicity and Response to Anti-PD-1 Blockade. Nat Commun (2019) 10:2782. doi: 10.1038/s41467-019-10669-0 31239444 PMC6592937

[16] Miao W Chen J Jia L Ma J Song D. The M6a Methyltransferase METTL3 Promotes Osteosarcoma Progression by Regulating the M6a Level of LEF1. Biochem Biophys Res Commun (2019) 516:719–25. doi: 10.1016/j.bbrc.2019.06.128 31253399

[17] Vengoechea J Sloan AE Chen Y Guan X Ostrom QT Kerstetter A. Methylation Markers of Malignant Potential in Meningiomas. J Neurosurg (2013) 119:899–906. doi: 10.3171/2013.7.JNS13311 23930849

[18] Hwang M Han MH Park HH Choi H Lee KY Lee YJ. LGR5 and Downstream Intracellular Signaling Proteins Play Critical Roles in the Cell Proliferation of Neuroblastoma, Meningioma and Pituitary Adenoma. Exp Neurobiol (2019) 28:628–41. doi: 10.5607/en.2019.28.5.628 PMC6844835 31698554

[19] Pitt JM Marabelle A Eggermont A Soria JC Kroemer G Zitvogel L. Targeting the Tumor Microenvironment: Removing Obstruction to Anticancer Immune Responses and Immunotherapy. Ann Oncol (2016) 27:1482–92. doi: 10.1093/annonc/mdw168 27069014

[20] Li YD Veliceasa D Lamano JB Lamano JB Kaur G Biyashev D. Systemic and Local Immunosuppression in Patients With High-Grade Meningiomas. Cancer Immunol Immunother (2019) 68:999–1009. doi: 10.1007/s00262-019-02342-8 31030234 PMC6531348

[21] Polyzoidis S Koletsa T Panagiotidou S Ashkan K Theoharides TC. Mast Cells in Meningiomas and Brain Inflammation. J Neuroinflamm (2015) 12:170. doi: 10.1186/s12974-015-0388-3 PMC4573939 26377554

[22] Chen X Tian F Lun P Feng Y. Profiles of Immune Infiltration and Its Relevance to Survival Outcome in Meningiomas. Biosci Rep (2020) 40(5):BSR20200538. doi: 10.1042/BSR20200538 32378707 PMC7225412

[23] Lu B Finn OJ. T-Cell Death and Cancer Immune Tolerance. Cell Death Differ (2008) 15:70–9. doi: 10.1038/sj.cdd.4402274 18007660

[24] Liu Y Cao X. Characteristics and Significance of the Pre-Metastatic Niche. Cancer Cell (2016) 30:668–81. doi: 10.1016/j.ccell.2016.09.011 27846389

[25] Corbet C Feron O. Tumour Acidosis: From the Passenger to the Driver's Seat. Nat Rev Cancer (2017) 17:577–93. doi: 10.1038/nrc.2017.77 28912578

[26] Li L Yu R Cai T Chen Z Lan M Zou T. Effects of Immune Cells and Cytokines on Inflammation and Immunosuppression in the Tumor Microenvironment. Int Immunopharmacol (2020) 88:106939. doi: 10.1016/j.intimp.2020.106939 33182039

[27] Tabernero MD Maillo A Gil-Bellosta CJ Castrillo A Sousa P Merino M. Gene Expression Profiles of Meningiomas are Associated With Tumor Cytogenetics and Patient Outcome. Brain Pathol (2009) 19:409–20. doi: 10.1111/j.1750-3639.2008.00191.x PMC8094742 18637901

[28] Patel AJ Wan YW Al-Ouran R Revelli JP Cardenas MF Oneissi M. Molecular Profiling Predicts Meningioma Recurrence and Reveals Loss of DREAM Complex Repression in Aggressive Tumors. Proc Natl Acad Sci USA (2019) 116:21715–26. doi: 10.1073/pnas.1912858116 PMC6815170 31591222

[29] Zhang B Wu Q Li B Wang D Wang L Zhou YL. M(6)A Regulator-Mediated Methylation Modification Patterns and Tumor Microenvironment Infiltration Characterization in Gastric Cancer. Mol Cancer (2020) 19:53. doi: 10.1186/s12943-020-01170-0 32164750 PMC7066851

[30] Steen CB Liu CL Alizadeh AA Newman AM. Profiling Cell Type Abundance and Expression in Bulk Tissues With CIBERSORTx. Methods Mol Biol (2020) 2117:135–57. doi: 10.1007/978-1-0716-0301-7_7 PMC7695353 31960376

[31] Jiao X Sherman BT Huang da W Stephens R Baseler MW Lane HC. DAVID-WS: A Stateful Web Service to Facilitate Gene/Protein List Analysis. Bioinformatics (2012) 28:1805–6. doi: 10.1093/bioinformatics/bts251 PMC3381967 22543366

[32] Liu J Eckert MA Harada BT Liu SM Lu Z Yu K. M(6)A mRNA Methylation Regulates AKT Activity to Promote the Proliferation and Tumorigenicity of Endometrial Cancer. Nat Cell Biol (2018) 20:1074–83. doi: 10.1038/s41556-018-0174-4 PMC6245953 30154548

[33] Liu L Wang J Sun G Wu Q Ma J Zhang X. M(6)A mRNA Methylation Regulates CTNNB1 to Promote the Proliferation of Hepatoblastoma. Mol Cancer (2019) 18:188. doi: 10.1186/s12943-019-1119-7 31870368 PMC6927193

[34] Du J Ji H Ma S Jin J Mi S Hou K. M6a Regulator-Mediated Methylation Modification Patterns and Characteristics of Immunity and Stemness in Low-Grade Glioma. Brief Bioinform (2021) 22(5):bbab013. doi: 10.1093/bib/bbab013 33594424

[35] Huang H Weng H Chen J. M(6)A Modification in Coding and Non-Coding RNAs: Roles and Therapeutic Implications in Cancer. Cancer Cell (2020) 37:270–88. doi: 10.1016/j.ccell.2020.02.004 PMC7141420 32183948

[36] Dong Z Cui H. The Emerging Roles of RNA Modifications in Glioblastoma. Cancers (Basel) (2020) 12(3):L736. doi: 10.3390/cancers12030736 PMC7140112 32244981

[37] Xu Y Ye S Zhang N Zheng S Liu H Zhou K. The FTO/miR-181b-3p/ARL5B Signaling Pathway Regulates Cell Migration and Invasion in Breast Cancer. Cancer Commun (Lond) (2020) 40:484–500. doi: 10.1002/cac2.12075 32805088 PMC7571404

[38] Chang G Shi L Ye Y Shi H Zeng L Tiwary S. YTHDF3 Induces the Translation of M(6)A-Enriched Gene Transcripts to Promote Breast Cancer Brain Metastasis. Cancer Cell (2020) 38:857–71.e857. doi: 10.1016/j.ccell.2020.10.004 33125861 PMC7738369

[39] Wang M Liu J Zhao Y He R Xu X Guo X. Upregulation of METTL14 Mediates the Elevation of PERP mRNA N(6) Adenosine Methylation Promoting the Growth and Metastasis of Pancreatic Cancer. Mol Cancer (2020) 19:130. doi: 10.1186/s12943-020-01249-8 32843065 PMC7446161

[40] Mathoux J Henshall DC Brennan GP. Regulatory Mechanisms of the RNA Modification M(6)A and Significance in Brain Function in Health and Disease. Front Cell Neurosci (2021) 15:671932. doi: 10.3389/fncel.2021.671932 34093133 PMC8170084

[41] Li Y Sheng H Ma F Wu Q Huang J Chen Q. RNA M(6)A Reader YTHDF2 Facilitates Lung Adenocarcinoma Cell Proliferation and Metastasis by Targeting the AXIN1/Wnt/beta-Catenin Signaling. Cell Death Dis (2021) 12:479. doi: 10.1038/s41419-021-03763-z 33980824 PMC8116339

[42] Sahm F Schrimpf D Stichel D Jones DTW Hielscher T Schefzyk S. DNA Methylation-Based Classification and Grading System for Meningioma: A Multicentre, Retrospective Analysis. Lancet Oncol (2017) 18:682–94. doi: 10.1016/S1470-2045(17)30155-9 28314689

[43] Sievers P Sill M Blume C Tauziede-Espariat A Schrimpf D Stichel D. Clear Cell Meningiomas Are Defined by a Highly Distinct DNA Methylation Profile and Mutations in SMARCE1. Acta Neuropathol (2021) 141:281–90. doi: 10.1007/s00401-020-02247-2 PMC7847462 33319313

[44] Zador Z Landry AP Saha A Cusimano MD. Gene Expression Signatures Identify Biologically Homogenous Subgroups of Grade 2 Meningiomas. Front Oncol (2020) 10:541928. doi: 10.3389/fonc.2020.541928 33224871 PMC7674612

[45] Williams EA Santagata S Wakimoto H Shankar GM Barker FG 2nd Sharaf R. Distinct Genomic Subclasses of High-Grade/Progressive Meningiomas: NF2-Associated, NF2-Exclusive, and NF2-Agnostic. Acta Neuropathol Commun (2020) 8:171. doi: 10.1186/s40478-020-01040-2 33087175 PMC7580027

[46] Huang P Guo YD Zhang HW. Identification of Hub Genes in Pediatric Medulloblastoma by Multiple-Microarray Analysis. J Mol Neurosci (2020) 70:522–31. doi: 10.1007/s12031-019-01451-4 31820345

[47] Pan Y Tong JHM Kang W Lung RWM Chak WP Chung LY. EXOSC4 Functions as a Potential Oncogene in Development and Progression of Colorectal Cancer. Mol Carcinog (2018) 57:1780–91. doi: 10.1002/mc.22896 30155936

