# Supplementary figures and images for: m6A Regulator Expression Segregates Meningiomas Into Biologically Distinct Subtypes
Source: Front Oncol. 2021 Dec 22;11:760892. doi: 10.3389/fonc.2021.760892 (PMC8727752; doi:10.3389/fonc.2021.760892)

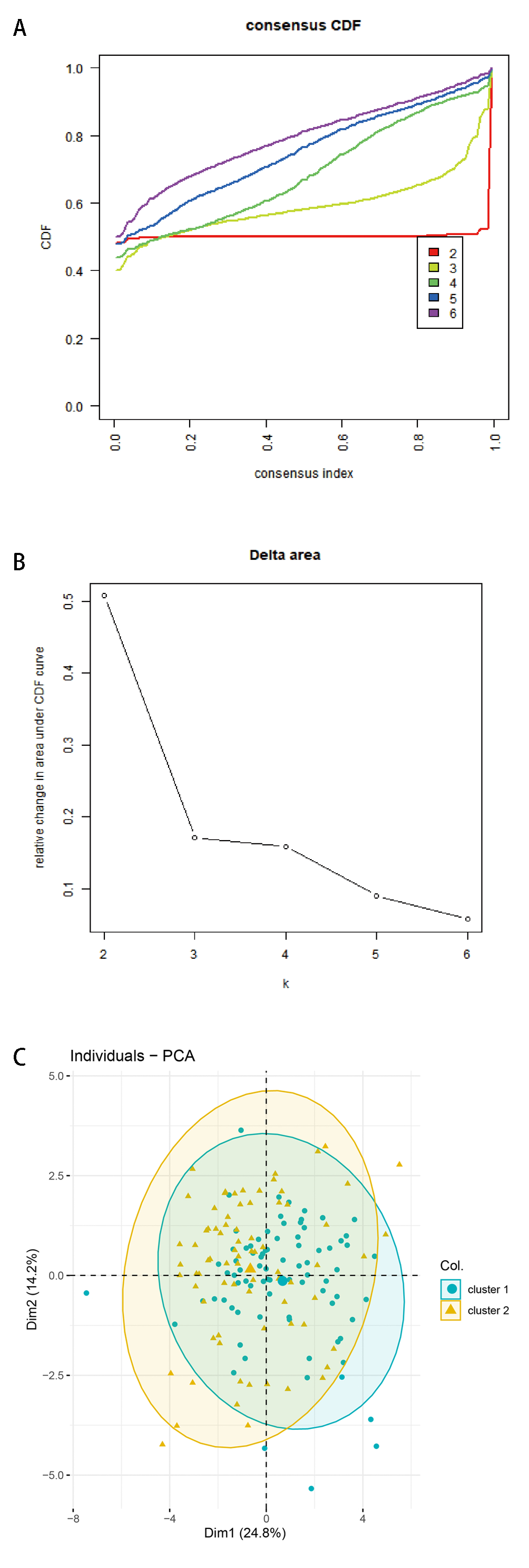

Supplement: Supplementary Figure 1 — (A, B) Best inflection point identified by the sum of squares for the error method. (C) Principal component analysis of two m6A clusters of the GSE136661 cohort. [file Image_1.tif]

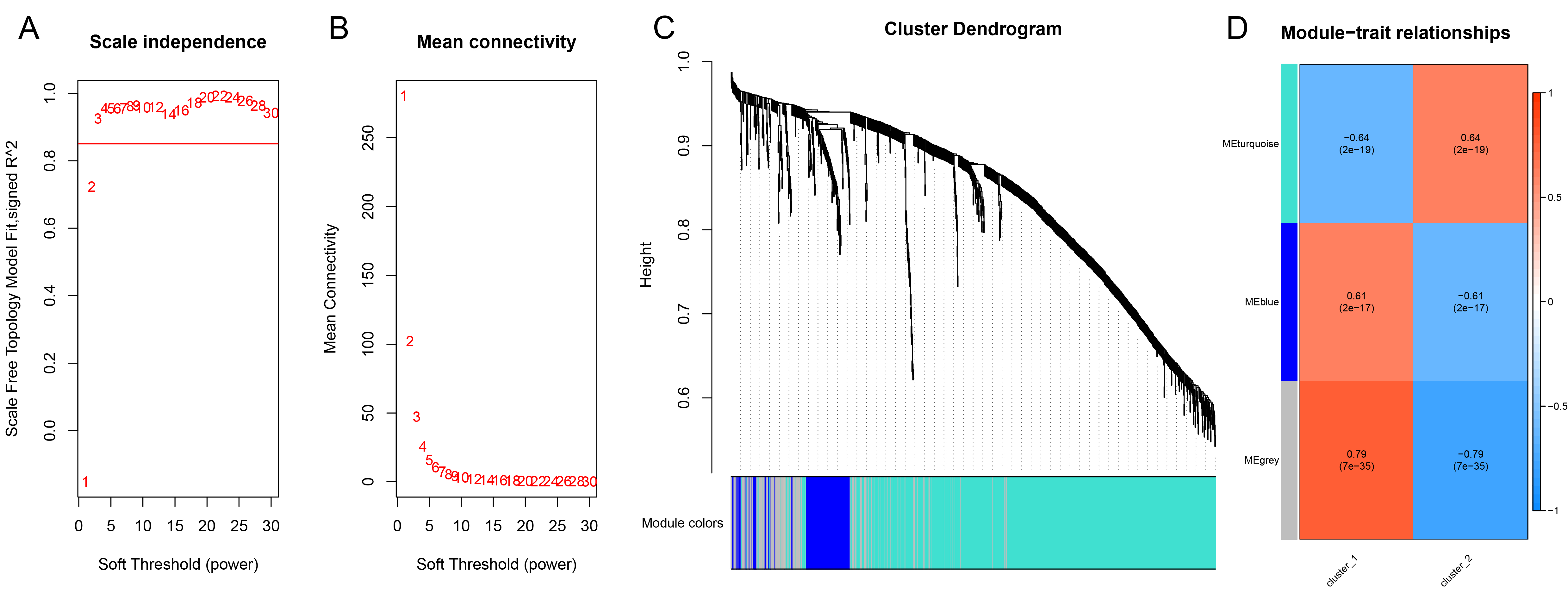

Supplement: Supplementary Figure 2 — (A, B) Analysis of network topology for various soft-thresholding powers. (C) Gene dendrogram and module colors. (D) Module–trait relationships of different modules and m6A clusters. [file Image_2.tiff]

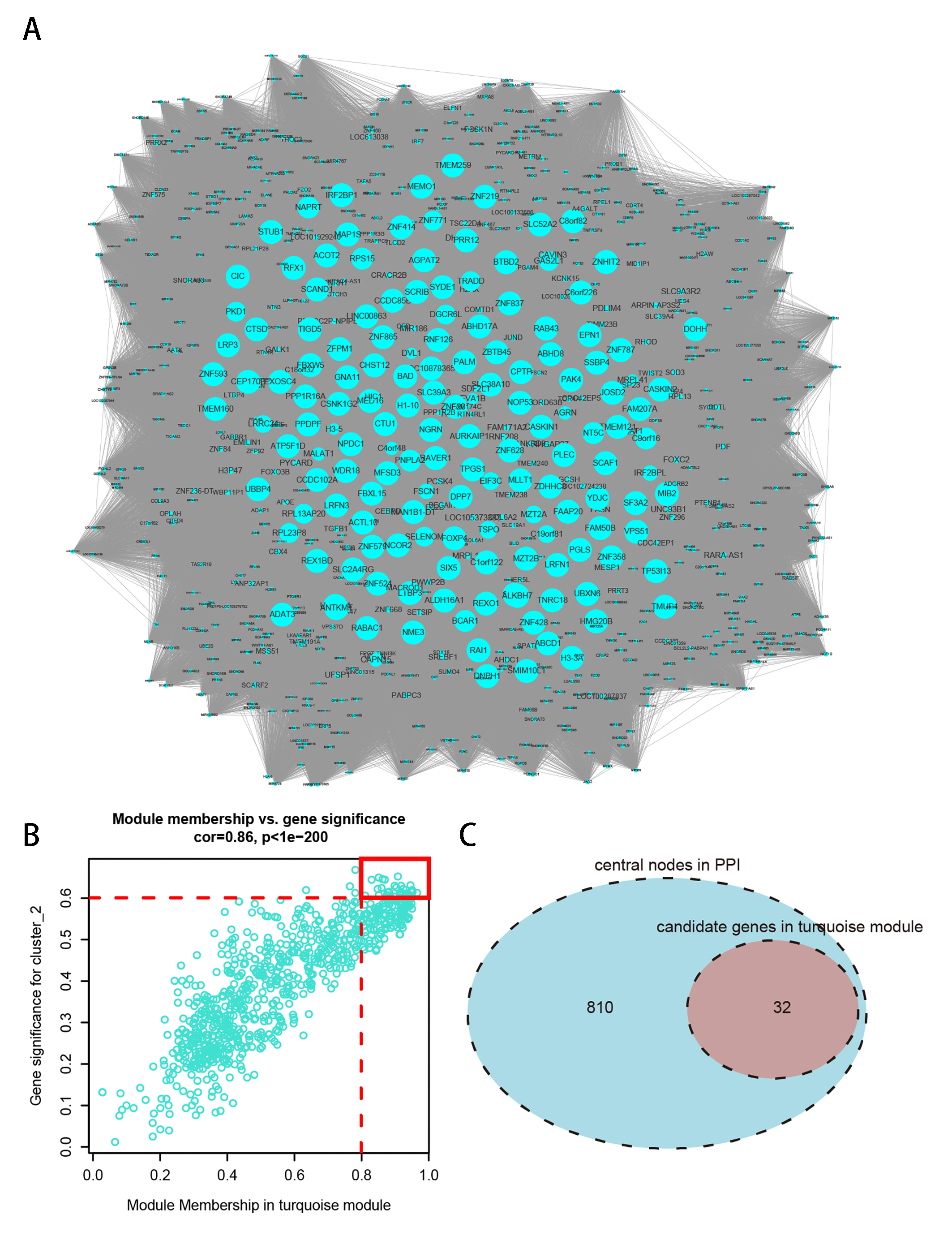

Supplement: Supplementary Figure 3 — (A) PPI network of the turquoise module obtained from the STRING database. (B) Correlation analysis of the turquoise module and m6A cluster characteristics. (C) Venn diagram of central nodes in PPI network and candidate genes in the turquoise module. [file Image_3.tiff]

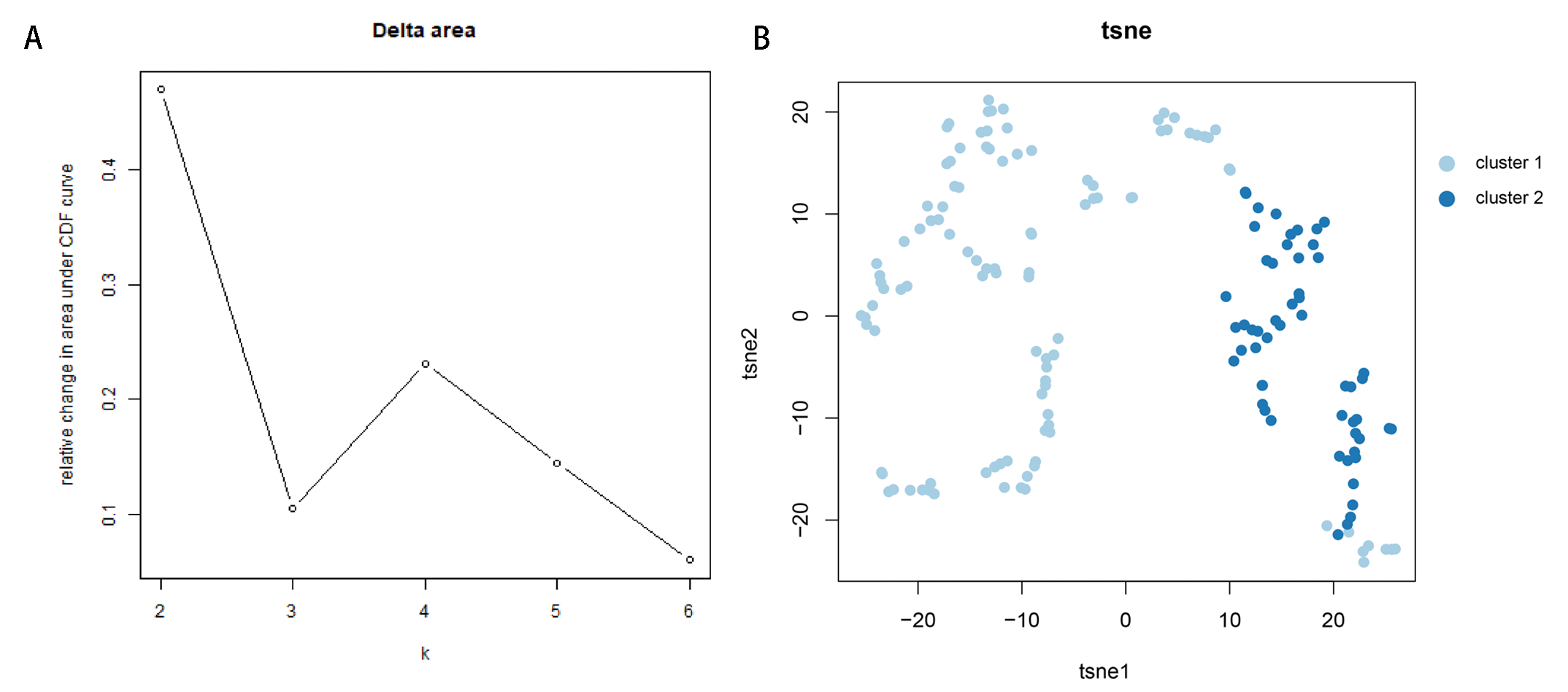

Supplement: Supplementary Figure 4 — (A) Best inflection point identified by the SSE method. (B) Dimensionality reduction and clustering of meningioma samples conducted by the t-SNE algorithm. [file Image_4.tif]
